# High tibial closing wedge osteotomy for medial compartment osteoarthrosis of knee

**DOI:** 10.4103/0019-5413.38585

**Published:** 2008

**Authors:** SM Tuli, Varun Kapoor

**Affiliations:** Department of Orthopedics, Vimhans Hospital, Nehru Nagar, New Delhi - 110 065, India

**Keywords:** Closing wedge osteotomy, genu varum, high tibial osteotomy, knee osteoarthrosis

## Abstract

**Background:**

Most patients of symptomatic osteoarthrosis of knee are associated with varus malalignment that is causative or contributory to painful arthrosis. It is rational to correct the malalignment to transfer the functional load to the unaffected or less affected compartment of the knee to relieve symptoms. We report the outcome of a simple technique of high tibial osteotomy in the medial compartment osteoarthrosis of the knee.

**Materials and Methods:**

Between 1996 and 2004 we performed closing wedge osteotomy in 78 knees in 65 patients. The patients selected for osteotomy were symptomatic essentially due to medial compartment osteoarthrosis associated with moderate genu varum. Of the 19 patients who had bilateral symptomatic disease 11 opted for high tibial osteotomy of their second knee 1-3 years after the first operation. Preoperative grading of osteoarthrosis and postoperative function was assessed using Japanese Orthopaedic Association (JOA) rating scale.

**Results:**

At a minimum follow-up of 2 years (range 2-9 years) 6-10° of valgus correction at the site of osteotomy was maintained, there was significant relief of pain while walking, negotiating stairs, squatting and sitting cross-legged. Walking distance in all patients improved by two to four times their preoperative distance of 200-400 m. No patient lost any preoperative knee function. The mean JOA scoring improved from preoperative 54 (40-65) to 77 (55-85) at final follow-up.

**Conclusion:**

Closing wedge high tibial osteotomy performed by our technique can be undertaken in any setup with moderate facilities. Operation related complications are minimal and avoidable. Kirschner wire fixation is least likely to interfere with replacement surgery if it becomes necessary.

## INTRODUCTION

Patients with osteoarthrosis of the knee predominantly due to medial compartment involvement have been treated by high tibial valgus osteotomy by various techniques. These include a closing wedge osteotomy, opening wedge osteotomy, dome osteotomy, hemicallotasis progressive corrective osteotomy and other modifications.^1^–^8^ The osteotomy has been fixed using staples, plates, external fixators, Kirschner wires, plaster casts and combinations.^9^–^13^ We report the outcome of a simple technique of high tibial osteotomy in the medial compartment osteoarthrosis of the knee.

## MATERIALS AND METHODS

Seventy eight knees in 65 patients with medial compartment osteoarthrosis and moderate genu varum between 1996 and 2004 were included in the present report. Patients selected were those walking independently or with one stick and who accepted the operation of “repairing their joint” rather than “replacing their joint”. The patients were explained the limitations of both the operations. Preoperative and postoperative rehabilitation exercises were demonstrated to the patient and the family. Non-walkers due to generalized arthropathies or due to medical comorbidities, flexion deformity of the knee of more than 10° or range of motion less than 90°, active rheumatoid arthritis or active infection, gross symptomatic lateral compartment involvement, more than 1 cm lateral subluxation of the tibia as judged in standing anteroposterior X-rays of both knees were considered unsuitable for high tibial osteotomy operation. However, there were patients who were more suitable for knee replacement surgery but they desired a repaired natural joint rather than an artificial prosthetic joint. High tibial osteotomy was done in such patients as an extended indication. In patients with bilateral symptomatic disease the one which was more advanced clinically and radiologically was operated first. Two patients underwent the osteotomy operation on both their knees in one sitting because they had favorable family support at home. Of the 19 patients with bilateral symptomatic disease 11 patients underwent high tibial osteotomy on their second knee 1-3 years after the first operation.

### Surgical procedure

Knee is kept flexed approximately at 90° throughout the operation. A lazy curved incision extending from lateral epicondyle of femur to the head of fibula and proceeding distally to lower extent of tibial tuberosity was made. The upper part of lateral surface of tibia was exposed subperiosteally and bleeds were cauterized. The upper end of fibula was exposed by subperiosteal reflection of lateral collateral ligament, insertion of biceps femoris and origin of peronei as a continuous flap (to be resutured to soft tissues after completion of operation). The superior tibio-fibular joint was opened. The medial half of fibular head was excised obliquely, resecting from superolateral to inferomedial direction. The oblique resection permits the remaining fibula to slide proximally while correcting the varus deformity. Common peroneal nerve lies medial to the distal part of the biceps femoris tendon from where it traverses distally to wind around the fibular neck. As a rule the peroneal nerve can be palpated and does not require exposure. Lateral surface of the tibia proximal to the tibial tubercle was exposed superiosteally. The muscles behind the upper part of the tibia were elevated subperiosteally, the posterior soft tissues were retracted posteriorly with the help of narrow bone levers. Flexed position of the knee joint and the posteriorly placed bone levers protect the soft tissues and popliteal vessels. A 15- to 20-cm long Kirschner wire was passed latero-medially through the knee joint space to give the orientation of the articular surface of proximal tibia. The osteotomy cuts were made with a small osteotome. Proximal osteotomy is made in the anterior half of tibia, parallel to the tibial joint line and 1.5-2 cm distal to it, posterior cortex at this level is not cut. Distal osteotomy is made 1-1.5 cm distal to the proximal cut, latero-medially in an oblique fashion to meet the proximal cut medially. The distal osteotomy cuts through lateral, anterior, posterior and medial tibial cortices and stays proximal to the insertion of ligamentum patellae. The proximal and distal cuts are connected by a vertical osteotomy cut made on the antero-lateral surface of tibia. Anterior wedge-shaped bone segment is removed, medial cortex-cuts are completed with a small osteotome. Osteotomy is completed by gentle valgus strain.

**Figure 1:**
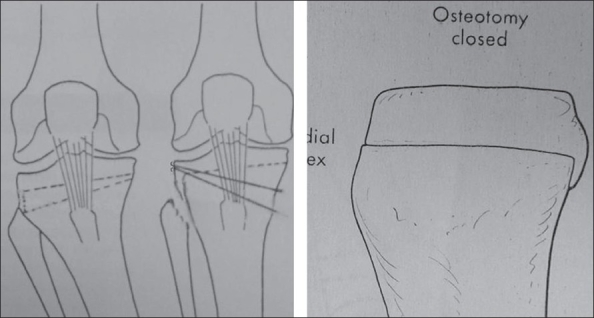
Diagrammatic representation of HTO. Note ledges of bone from the lateral and posterior cortices of proximal segment

**Figure 2:**
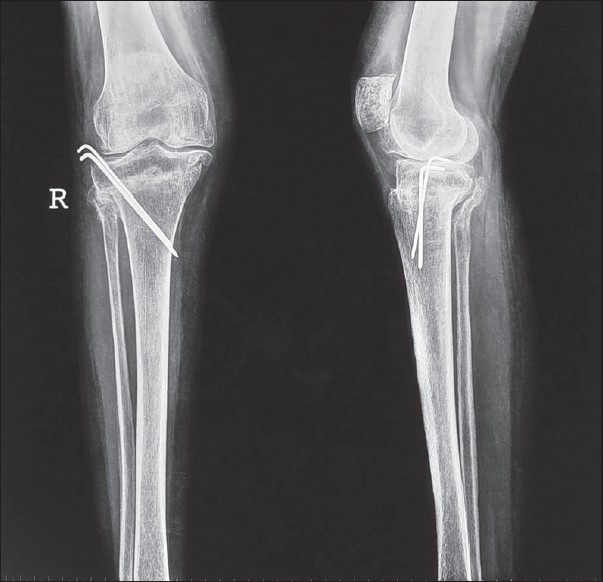
X-rays soon after HTO showing K wires and lateral and posterior ledges from the proximal segment. Note slight anterior positioning of tibial tubercle from the distal fragment

A small amount of cancellous bone from the lateral aspect of proximal fragment was removed. One can now appreciate two ledges of cortical bone [Figures 1–2] from the proximal wider segment of tibia, one situated posteriorly (made by posterior cortex) and the other situated laterally (made by lateral cortex). On completion of the osteotomy the distal narrower segment of tibia is telescoped in the wider proximal segment. The posterior and lateral ledges of bone from the proximal segment add some stability at the site of osteotomy and seating of the proximal end of distal fragment anterior to the posterior ledge of bone from the proximal fragment probably relaxes the ligamentum patellae by a few millimeters. Having obtained the desired correction of 7° valgus and 5° of external rotation, two Kirschner's (K) wires (usually 2-mm thick) are passed from the superolateral part of tibia crossing the site of osteotomy to engage the medial cortex of the distal fragment for further stability [Figure 2]. The bones harvested during the operation are used as bone grafts and packed around the site of osteotomy. The wound is closed in layers over a suction drain. The limb is put in a plaster cast from mid-thigh to the ankle.

The stitches are removed at three to 4 weeks after the operation and a well-molded plaster cylinder cast is applied ensuring 7° of valgus, 5° of external rotation at the osteotomy site and 5° of knee flexion. The semirigid fixation provided by two K-wires permit minor corrections to achieve the best desired clinical position of the limb while applying the plaster. Ambulation with toe-touch is encouraged three to 4 days after the operation with a walker or two crutches. Full loading on the operated limb is encouraged 3-4 weeks after the operation with a single crutch in the contralateral hand. The plaster cast is usually removed three to three and a half months after the operation, full weight-bearing with a single crutch is encouraged and range of motion active exercises are begun. K-wires are removed 6-12 months after the osteotomy.

This is an analysis of 65 patients who underwent high tibial osteotomy (HTO) on 78 knees. Eleven patients had osteotomy on both knees at an interval of 1-3 years (after the operation on the first knee), these patients were satisfied with the outcome of the first osteotomy; and two had osteotomy on both knees simultaneously in the same sitting (these patients desired simultaneous HTO on both sides because they had favorable family support at home). The age at operation ranged from 56-73 years, there were 19 males and 46 females. The patients were fully informed about the details of the postoperative regime and rehabilitation and the expectation of the outcome in their language.The evaluation was done according to Japanese Orthopaedic Association score [Table 1]. The mean pre-operation JOA score was 54 (40-65). Of the 78 operated knees the radiological staging (on X-rays in standing position) prior to HTO according to the Japanese Orthopaedics Association system was as follow:^14^

**Table 1:** Knee rating scale for clinical evaluation of osteoarthr it is of the knee (Japanese Orthopaedic Association – Yasuda 1992)

Pain (30 points)	
No pain at any time	30
Mild starting pain	20
Moderate pain on walking	10
Severe pain on walking	5
Severe pain at rest	0
Function (20 points)	
Walking unlimited	20
Walking distance of 0.5 to 1 km	15
Walking less than 0.5 km	10
Walking only indoors	5
Cannot walk	0
Range of motion (20 points)	
More than 120°	20
119°-90°	15
89°-60°	10
59°-30°	5
Less than 30°	0
Flexion deformity (10 points)	
0°-10°	10
11°-30°	5
More than 30°	0
Varus or valgus deformity (10 points)	
Less than 5°	10
6°-15°	5
More than 15°	0
Activities of daily living (10 points)	
Rising from chair	
Climbing stairs	
Going downstairs	
One foot standing	
Running	


(2 points for easy; 1 point, difficult; 0 points, impossible in each item)

### Radiological stages

1. Bony spur only = None
2. Narrowing of medial joint space (less than half of normal) = 24
3. Narrowing of medial joint space (more than half of normal) = 26
4. Obliteration of medial joint space with minor bone erosion = 18
5. Major bone erosion with lateral subluxation (up to 1 cm) = 10

## RESULTS

**Figure 3:**
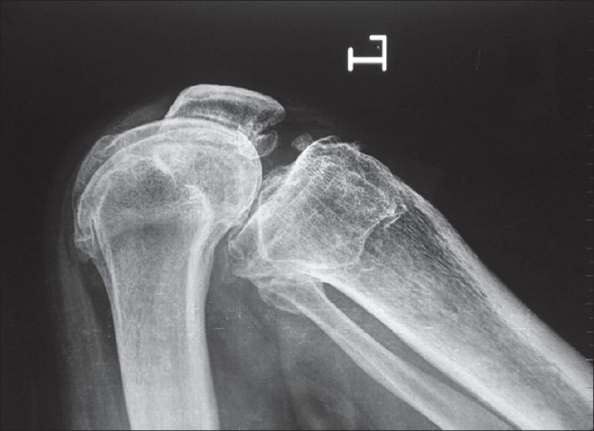
This patient was operated at 64 years of age. Now at 73 years of age, 9 years after HTO the patient has full range of knee flexion

**Figure 4:**
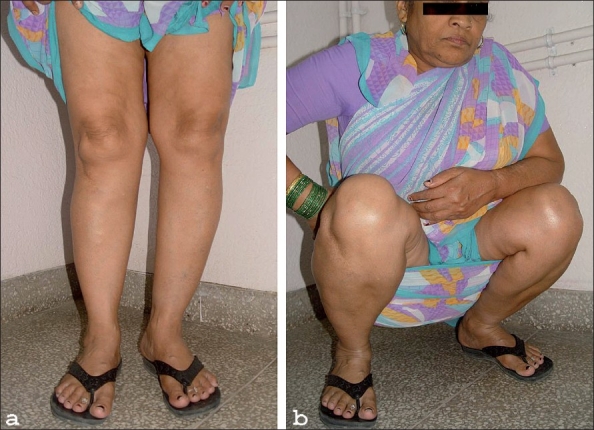
Clinical and functional result of the HTO done on right knee, 6 years after operation

Follow-up analysis was done on those knees which were available for clinical and radiological examination at 2 years or more after the HTO procedure. Eighty per cent (*n* = 62) patients perceived relief of pain by 75% or more, in the remaining 16 there was relief of pain by 40-70%. There was no patient at 2 years follow-up who felt no relief of pain or had exacerbation of pain. None of the patients [Figures 3–4] has lost any function like range of motion, capability of squatting and cross-leg sitting, as compared to their preoperative status. There was not much difference between the preoperative and postoperative range of movements of the knees. The postoperative range varied from 90 to 145° (mean 135°). The distance the patients were able to walk without significant pain prior to operation varied from 60 m to 1 km, the walking distance without significant pain improved postoperatively to 1-5 km (mean 3 km). There was marked improvement in the pattern of negotiating stairs and in the lurch while walking. According to the total JOA scores [Table 1] the mean score improved from 54 (40-65) before operation to 77 (55-85) at the final follow-up. The least gratifying results were observed in patients who were in radiological Stage 5 at the time of HTO.

Postoperative standing X-ray of both knees at 2 years showed maintenance of the valgus from 5° to 10°. No significant increase in the medial joint space height was observed. The radiological staging 2 years after the operation remained unchanged. Complications observed in 78 HTO cases were foot drop in two ladies: one recovered spontaneously in 6 months time, one did not recover up to 12 months when tendon transfer for foot drop was performed. Exploration of the common peroneal nerve at that time did not reveal any break in the continuity of the nerve. One male patient had delayed union which healed by loading in a knee brace by 5 months after HTO. Superficial infection and breakdown of the operative wound occurred in one lady with medical comorbidities. The infection healed by dressing and antibiotics.

## DISCUSSION

Medial compartment osteoarthrosis of knee associated with genu varum deformity should be considered a malalignment contributing to degenerative changes. It is rational to correct malalignment which would transfer the load to the less affected compartment of the knee to relieve clinical symptoms and hopefully slow down the progression of degeneration.^15^,^16^ We do not expect reversal or halting of osteoarthritic changes in the operated knee joints as these are natural age-related changes. We however did not observe accelerated degenerated change in the operated knee joints as compared to the changes in the contralateral unoperated knees during a follow-up of 2-9 years.

Our study cannot make a statement regarding the advantage or otherwise of using a closing wedge osteotomy as compared to an opening wedge osteotomy, however, review of the literature favors a closing wedge osteotomy.^1^–^7^,^16^–^21^ Closing wedge osteotomy as performed by our technique is through the cancellous bone that would minimize chances of delayed union or nonunion. Closing wedge HTO is an excellent operative option with a long experience of success with minimum chances of nonunion.^1^–^7^,^16^–^18^ The osteotomy close to the joint line ensured more accurate correction of deformity.

Many workers have used elaborate implants to fix the osteotomy, a review of their results, however, do not reflect any exceptional advantage in the clinical outcome. Less extensive implants would probably cause least disturbance for any future replacement procedures.^8^–^10^,^12^,^16^,^22^,^23^ We have used a non-rigid fixation with two K-wires, however posterior and lateral ledges of bone from the proximal segment of tibia added some stability at the site of osteotomy. We supplemented stability for loading using a postoperative plaster cast. The first change of plaster (always done by the surgeon) after stitch removal was done around 3 weeks after the operation. The semirigid fixation permitted finer corrections of the position and immobilization in the best desired position of 7° of valgus and 5° of external rotation at the site of osteotomy and 5° of flexion at the knee joint.

The importance of adequate correction and maintenance of valgus alignment has been emphasized by many workers to achieve optimal clinical outcome.^1^,^17^,^18^,^20^ The follow-up of our patient 2-9 years after osteotomy did not show reversal to a varus deformity in any case.

Factors associated with less favorable results are obesity (more than 30% of the ideal weight) and severe limitation of motion before surgery.^24^ High tibial osteotomy provides an alternative to unicompartmental replacement or total knee arthroplasty in selected patients. When properly performed, HTO should not much compromise later arthroplasty if it becomes necessary. The patient can achieve appreciable relief of pain lasting for 10-15 years with normal proprioception and with no drastic restriction of preoperative activities.^24^–^29^

## References

[1] Conventry MB (1965). Osteotomy of the upper portion of the tibia for degenerative arthritis of the knee: A preliminary report. J Bone Joint Surg Am.

[2] Conventry MB (1984). Upper tibial osteotomy. Clin Orthop Relat Res.

[3] Conventry MB (1985). Upper tibial osteotomy for osteoarthritis. J Bone Joint Surg Am.

[4] Conventry MB, Hstrup DM, Wallrichs SL (1993). Proximal tibial osteotomy: A critical long-term study of eighty-seven cases. J Bone Joint Surg Am.

[5] Hernigou P, Medevielle D, Debeyre J, Goutallier D (1987). Proximal tibial osteotomy for osteoarthritis with varus deformity: A ten to thirteen year followup study. J Bone Joint Surg Am.

[6] Magyar G, Ahl TL, Vibe P, Toksvig-Larsen S, Lindstrand A (1999). Open-wedge technique for osteoarthritis of the knee: A randomized study of 50 operations. J Bone Joint Surg Br.

[7] Sundaram NA, Hallett JP, Sullivan MF (1986). Dome osteotomy of the tibia for osteoarthritis of the knee. J Bone Joint Surg Br.

[8] Klinger HM, Lorenz F, Harer T (2001). Open wedge tibial osteotomy by hemicallotasis for medial compartment osteoarthritis. Arch Orthop Trauma Surg.

[9] Koshino T, Morii T, Wada J, Saito H, Ozawa N, Noyori K (1989). High tibial osteotomy with fixation by a blade plate for medial compartment osteoarthritis of the knee. Orthop Clin North Am.

[10] Geiger F, Schneider U, Lukoschek M, Ewerbeck V (1999). External fixation in proximal tibial osteotomy: A comparison of three methods. Int Orthop.

[11] Billings A, Scott DF, Camargo MP, Hofmann AA (2000). High tibial osteotomy with a calibrated osteotomy guide, rigid internal and early motion: Long-term followup. J Bone Joint Surg Am.

[12] Weale AE, Lee AS, Maceachern AG (2001). High tibial osteotomy using a dynamic axial external fixator. Clin Orthop Relat Res.

[13] Amendola A, Panarella L (2005). High tibial osteotomy for the treatment of unicompartmental arthritis of the knee. Orthop Clin North Am.

[14] Yasuda K, Majima T, Suchida T, Kaneda K (1992). A ten to fifteen year followup observation of high tibial osteotomy in medial compartment osteoarthritis. Clin Orthop Relat Res.

[15] Goutallier D, Stephane Van, Driessche SV, Manicom O, Ali ES, Bernageau J (2006). Influence of lower-limb torsion on long-term outcomes of tibial valgus osteotomy for medial compartment knee osteoarthritis. J Bone Joint Surg Br.

[16] Maquet PV (1976). Valgus osteotomy for osteoarthritis of the knee. Clin Orthop Relat Res.

[17] Aglietti P, Rinonapoli E, Stringa G, Tavinani A (1983). Tibial osteotomy for the varus osteoarthritic knee. Clin Orthop Relat Res.

[18] Stuart MJ, Grace JN, Ilstrup DM, Kelly CM, Adams RA, Morrey BF (1990). Late recurrence of varus deformity after proximal tibial osteotomy. Clin Orthop Relat Res.

[19] Koshimo T, Tsuchiya K (1979). The effect of high tibial osteotomy on osteoarthritis of the knee. Int Orthop.

[20] Nguyen C, Rudan J, Simurda MA, Cooke TD (1989). High tibial osteotomy compared with high tibial and Maquet procedures in medial and patellofemoral compartment osteoarthritis. Clin Orthop Relat Res.

[21] Aoki Y, Yasuda K, Mikami S, Ohmoto S, Majima T, Minami A (2006). Inverted V-shaped high tibial osteotomy compared with closing-wedge high tibial osteotomy for osteoarthritis of knee. J Bone Joint Surg Br.

[22] Catagni MA, Guerreschi F, Ahmad TS, Cattaneo R (1994). Treatment of genu varum in medial compartment osteoarthritis of the knee using the Ilizarov method. Orthop Clin North Am.

[23] Brouwer RW, Bierma- Zeinstra SM, Raaij TM, Ivan Verhaar JA (2006). Osteotomy for medial compartment arthritis of the knee using a closing wedge 07 or an opening wedge controlled by a Puddu plate. J Bone Joint Surg Br.

[24] Naudie D, Bourne RB, Rorabeck CH, Bourne TJ (1999). The Install award: survivorship of the high tibial valgus osteotomy: A 10 to 22 years followup study. Clin Orthop Relat Res.

[25] Levy M, Pauker M, Lotem M, Seelenfreund M, Fried A (1973). High tibial osteotomy: A followup study and discussion of a modified technic. Clin Orthop Relat Res.

[26] Tjornstrand BA, Egund N, Hagstedt BV (1981). High tibial osteotomy a seven years clinical and radiographic followup. Clin Orthop Relat Res.

[27] Insall JN, Joseph DM, Miska C (1984). High tibial osteotomy for varus gonarthrosis: A long-term followup study. J Bone Joint Surg Am.

[28] Ivarsson L, Myrnerts R, Gillquist J (1990). High tibial osteotomy for medical osteoarthritis of the knee: A 5 to 7 and 11 yrs followup. J Bone Joint Surg Br.

[29] Rinonapoli E, Mancini GB, Corvaglia A, Musiello S (1998). Tibial osteotomy for varus gonarthrosis: A 10 to 21 years follouwp study. Clin Orthop Relat Res.

